# UK prescribing practices as proxy markers of unmet need in allergic rhinitis: a retrospective observational study

**DOI:** 10.1038/npjpcrm.2016.33

**Published:** 2016-06-23

**Authors:** David B Price, Glenis Scadding, Claus Bachert, Hesham Saleh, Shuaib Nasser, Victoria Carter, Julie von Ziegenweidt, Alice M S Durieux, Dermot Ryan

**Affiliations:** 1 University of Aberdeen, Aberdeen, UK; 2 Research in Real Life, Oakington, Cambridge, UK; 3 Observational and Pragmatic Research Institute Pte Ltd, Singapore, SG; 4 The Royal National Throat, Nose and Ear Hospital, London, UK; 5 Upper Airways Research Laboratory, Ghent University Hospital, Ghent, Belgium; 6 Imperial College NHS Healthcare, Charing Cross Hospital, London, UK; 7 Addenbrooke’s Hospital, Cambridge, UK; 8 Optimum Patient Care Ltd, Cambridge, UK; 9 Allergy and Respiratory Research Group, Usher Institute of Population Health Sciences and Informatics, University of Edinburgh, Edinburgh, UK

## Abstract

Little data on UK prescribing patterns and treatment effectiveness for allergic rhinitis (AR) are available. We quantified unmet pharmacologic needs in AR by assessing AR treatment effectiveness based on the prescribing behaviour of UK general practitioners (GP) during two consecutive pollen seasons (2009 and 2010). We conducted a retrospective observational study with the data from the Optimum Patient Care Research Database. We assessed diagnoses and prescription data for patients with a recorded diagnosis of rhinitis who took rhinitis medication during the study period. We assessed the data from 25,069 patients in 2009 and 22,381 patients in 2010. Monotherapy was the initial prescription of the season for 67% of patients with seasonal AR (SAR) and 77% of patients with nonseasonal upper airways disease (NSUAD), for both years. Initial oral antihistamine (OAH) or intranasal corticosteroid (INS) monotherapy proved insufficient for >20% of SAR and >37% of NSUAD patients. Multiple therapy was the initial prescription for 33% of SAR and 23% of NSUAD in both years, rising to 45% and >50% by season end, respectively. For NSUAD, dual-therapy prescriptions doubled and triple-therapy prescriptions almost tripled during both seasons. Many patients revisited their GP regardless of initial prescription. Initial OAH or INS monotherapy provides insufficient symptom control for many AR patients. GPs often prescribe multiple therapies at the start of the season, with co-prescription becoming more common as the season progresses. However, patients prescribed multiple therapies frequently revisit their GP, presumably to adjust treatment. These data suggest the need for more effective AR treatment and management strategies.

## Introduction

As a disease, allergic rhinitis (AR) is one of the most under-estimated diseases, in terms of its impact, severity, treatment and cost. More high-profile diseases, such as asthma, diabetes and heart disease, have been well quantified in terms of their impact, but not AR. AR affects ~600 million individuals in Europe^1^ and ~90 million in the United States.^2^ It has a substantial negative impact on patients’ lives if uncontrolled,^1,3,4^ with high associated costs, particularly indirect ones,^5,6^ which could be reduced by effective symptom control.

From the patient perspective, having symptomatic AR means living with any or all of the symptoms of nasal congestion, headache, postnasal drip, repeated sneezing, runny nose and other symptoms on a near-daily basis.^7–9^ Ocular symptoms are common, difficult to control and have the greatest negative impact on patients’ quality of life.^10,11^ AR symptoms impair patients’ function in day-to-day life,^4,12,13^ and cause sleep disturbance,^14^ fatigue, absenteeism and productivity loss at work and school (presenteeism).^3,15,16^ For those with co-morbid asthma, the presence of significant AR also predicts poor asthma control.^17^

From a physician's perspective, AR is becoming more challenging to diagnose and treat. The majority of AR patients attending clinic have moderate/severe disease,^11,18^ with most of them first visiting their doctor when their AR symptoms become ‘intolerable’.^19^ Data on sensitisation are often limited and conflicting, and patients are commonly poly-sensitised,^4,20,21^ making allergen avoidance problematic. Many also suffer from both allergic and non-allergic disease,^22^ and up to one-fifth of the patients are resistant or unresponsive to guideline-directed therapy.^23^ Furthermore, 10–11% of the UK and European population suffers from chronic rhinosinusitis, which may present overlapping symptoms.^24,25^ Finally, many physicians underestimate AR severity and consequently fail to issue adequate treatment,^26,27^ a situation that appears to have changed little in a decade.^28^

The Allergic Rhinitis and its Impact on Asthma (ARIA) Guideline proposes a stepwise approach to AR management depending on the severity and duration of symptoms,^1,29^ with the ultimate aim of symptom control. Intranasal steroids (INS) are recommended as first-line treatment for moderate/severe AR, both intermittent and persistent, and are currently considered the most effective medication class.^1,29,30^ ARIA currently states that there are insufficient data available to make a recommendation concerning the combined use of oral antihistamines (OAH) and INS,^1^ with most of the published literature showing no benefits gained by adding other AR treatments to INS therapy.^31,32^

The aim of this retrospective study was to quantify the unmet medical need in seasonal and perennial AR, specifically from the UK payer perspective. This unmet need was quantified using data on AR prescription to assess AR therapy failure, shift towards multiple therapy prescriptions and multiple General Practitioner (GP) consultations, all of which are proxy measures of symptom control, during two consecutive pollen seasons. To our knowledge, this is the first study to provide quantitative data on these prescribing patterns in the UK.

## Results

### Baseline characteristics

GP prescription data from 25,069 patients were assessed in 2009 and from 22,381 patients in 2010 (Table 1). The average age was about 30 years for seasonal AR (SAR) and about 47 years for nonseasonal upper airways disease (NSUAD) patients. Approximately one-third of SAR patients and over half of NSUAD patients had co-morbid asthma. About one-third of patients in both groups had an eczema diagnosis; co-morbid diagnoses of urticaria and nasal polyps were rare (<10% and <5% of patients, respectively). Most of the patients were non-smokers (Table 1).

### Initial recorded prescription of the season

Most patients commenced the season on a single AR therapy (SAR: 67%; NSUAD: 77%), with similar figures noted in 2009 and 2010 (Table 2). OAH monotherapy was the most common initial prescription, prescribed to over half of the patients, regardless of phenotype. INS, the second most commonly prescribed monotherapy, was more frequently prescribed to NSUAD patients (2009/2010 SAR: 8.0/7.2%; NSUAD: 15.3/16.6%) as initial therapy. However, a significant proportion (particularly in SAR patients) commenced the season on multiple therapy, with a similar incidence of co-prescribing behaviour in 2009 (SAR: 33.0%; NSUAD: 23.0%) and 2010 (SAR: 33.5%; NSUAD: 23.1%). OAH+INS was the most common multiple therapy regimen prescribed at the beginning of the season for both groups (2009/2010 SAR: 11.7/11.6%; NSUAD 10.1/10.0%). Eye drops were more commonly co-prescribed with OAH for SAR (2009/2010: 8.9/9.1%) than for NSUAD patients (2009/2010: 3.6/3.7%). Similarly, the triple-therapy regimen of OAH+INS+eye drops was prescribed more frequently at the start of the season to SAR (2009/2010: 8.9/9.5%) than to NSUAD patients (2009/2010: 3.0/2.9%; Table 2).

### Treatment outcomes for AR patients who commenced the season on monotherapy

**Figure 1:**
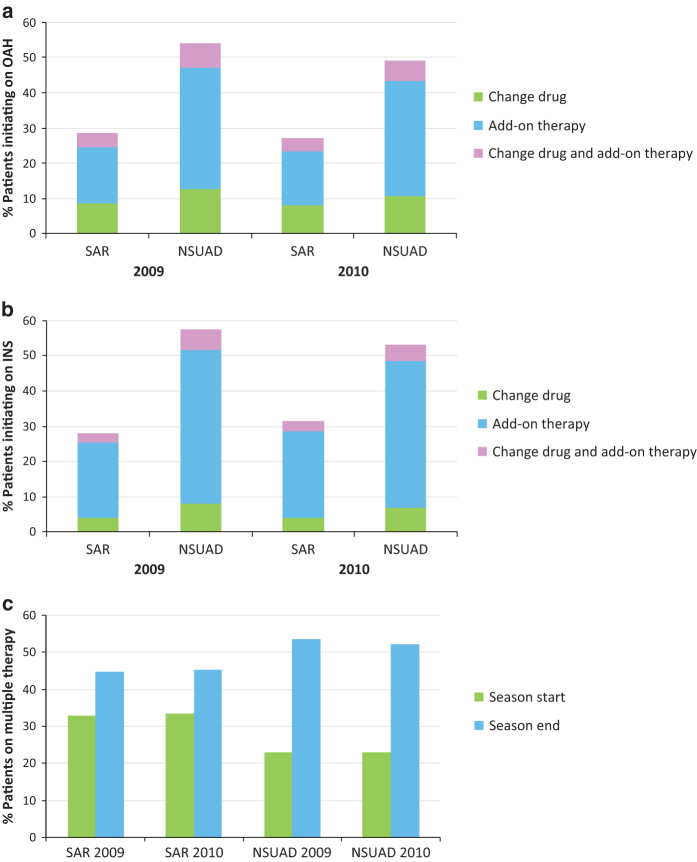
Prescription outcome for patients who started the season on monotherapy, with either (**a**) oral antihistamine (OAH) or (**b**) intranasal corticosteroid (INS) and (**c**) the proportion of seasonal allergic rhinitis (SAR) and nonseasonal upper airways disease (NSUAD) patients prescribing multiple therapies at the start and end of the season, for the 2009 and 2010 seasons. 2009—SAR: *n*=12,289; NSUAD: *n*=5,181. 2010—SAR: *n*=10,766; NSUAD: *n*=4,764. For **c**: 2009—SAR: *n*=18,341; NSUAD: *n*=6,728; 2010—SAR: *n*=16,187; NSUAD: *n*=6,194.

For SAR patients who started the 2009 season on OAH monotherapy, 8.8% of them changed OAH (within the same class), 15.7% added on a new therapy and 4.1% changed OAH and added on a new therapy (Figure 1a; Supplementary Table S1). A similar pattern was observed for the 2010 season. Treatment change following initial OAH prescription was more apparent in the NSUAD patients with a change of OAH noted in 12.6% of patients, therapy add-on in 34.5% and both OAH change and add-on in 7.0% of patients during the 2009 season, with similar findings recorded in 2010 (Figure 1a; Supplementary Table S1).

Treatment change following initial INS prescription also commonly occurred for both SAR and NSUAD patients. For SAR patients, GPs changed the INS (within class; 2009: 4.0%; 2010: 4.1%), added on to the initial INS monotherapy (2009: 21.3%; 2010: 24.6%), or changed INS and added on (2009: 2.8%; 2010; 2.8%; Figure 1b; Supplementary Table S1). Again, treatment changes following initial INS monotherapy prescription were more apparent for NSUAD patients. Doctors either changed INS (2009; 8.2%; 2010: 7.0%), added on to the INS (2009; 43.6%; 2010; 41.4%) or both (2009: 5.7%; 2010: 4.8%) (Figure 1b; Supplementary Table S1).

For all patients who added on therapies, >72% added a single therapy, >24% added two and >2% added three, regardless of whether they started on an OAH or an INS. For patients adding therapies, those who initially received an OAH monotherapy prescription were most commonly prescribed INS add-on (2009: 56.5%; 2010: 55.0%). Those who initially received INS monotherapy prescription were most commonly prescribed an OAH add-on (2009; 77.1%; 2010; 77.6%). Eye drops were a more common therapy add-on for patients who started the season on OAH monotherapy (2009: 26.0%; 2010: 27.2%) than for patients starting the season on INS monotherapy (2009: 10.6%; 2010: 9.5%; Supplementary Table S1).

### Dynamics of prescription change during the season

There was a shift to multiple therapy prescription during both seasons for both SAR and NSUAD. The proportion of SAR patients prescribed multiple therapy increased from 33% at season start to 45% at season end for both years (Table 3; Figure 1c). This shift was even more apparent for NSUAD, where the proportion on multiple therapy doubled from 23% at season start to 53.5% and 52.0% at season end for 2009 and 2010, respectively (Table 3; Figure 1c). For these NSUAD patients, the proportion on dual therapy doubled over the 2009 and 2010 seasons, from 19% at start to ≈40% at end, and the number on triple therapy almost tripled during both seasons, from 4% at start to 11.2% at end (Table 3).

### Multiple therapy partners at season end

The most common multiple therapy regimen at season end was OAH+INS for both SAR and NSUAD patients (Table 3). The proportion prescribed this dual therapy was slightly higher for NSUAD patients (2009: 21.5%; 2010: 20.6%) than for SAR patients (2009: 16.0%; 2010: 16.0%). Adding eye drops to either OAH monotherapy or OAH+INS dual therapy was the next most common prescribing option in both groups for both seasons; OAH+eye drops (SAR: ≈11%; NSUAD: ≈7%); OAH+INS+eye drops (SAR: ≈11%; NSUAD: ≈7%) (Table 3).

### General practice consultations

**Figure 2:**
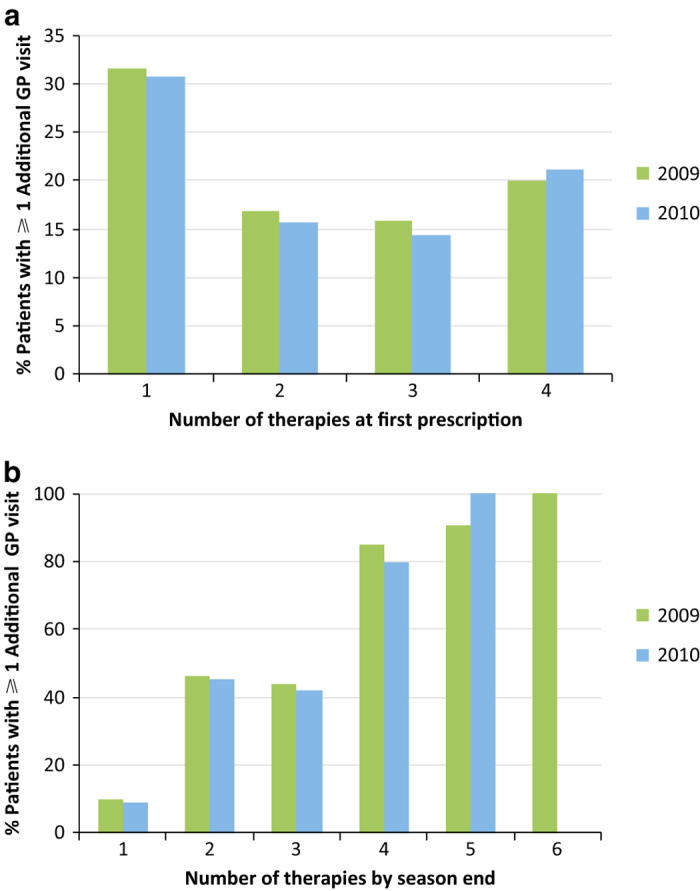
Proportion of allergic rhinitis (AR) patients requiring multiple GP consultations during the 2009 and 2010 seasons according to (**a**) number of therapies at first prescription and (**b**) number of therapies by season end. Season was defined as 1 March to 31 August for both years. 2009: *n*=25,069; 2010: *n*=22,381.

Many patients required an additional GP consultation after the initial consultation of the season. Almost one-third of patients (2009: 31.5%; 2010: 30.7%) who started the season on a monotherapy had an additional GP consultation (Figure 2a). Most of them incurred one additional visit (2009: 22.6%; 2010; 22.5%), but some reconsulted twice (2009: 6.4%; 2010: 6.1%) or even three times (2009: 1.8%; 2010: 1.3%). Patients who started the season on dual therapy also frequently reconsulted their GP (2009: 16.9%; 2010: 15.7%). Similar figures were seen for those who commenced the season on three and even four therapies (Figure 2a).

As the number of therapies prescribed during the season increased, so did the number of GP consultations (Figure 2b). Of patients who had received two therapies by the end of the season, 46.3% in 2009 and 45.5% in 2010 reconsulted their GP. The number of reconsultations was similar for patients prescribed three AR therapies over the season (2009: 44.1%; 2010: 42.2%). Of patients who had received four AR therapies during the season, 85.0% in 2009 and 79.3% in 2010 reconsulted, with most revisiting two (2009: 33.1%; 2010: 22.1%) and three times (2009: 30.0%; 2010: 23.6%).

## Discussion

### Main findings

This study provides a comprehensive view of AR therapy failure and co-prescribing behaviour among GPs in the UK during two separate seasons and according to AR phenotype (i.e., SAR and NSUAD). It provides data on how AR patients are treated in a real-world setting, in terms of initial prescription(s), treatment outcomes for patients with monotherapy failure, shifts to co-prescribing practices, most common multi-therapy regimens and the need for repeated GP consultations as a function of both initial and overall prescriptions during the season.

The survey found that the majority of patients, both SAR and NSUAD, received a monotherapy as their initial prescription of the season, but that this monotherapy (whether OAH or INS) proved to be insufficient for many, necessitating additional GP consultations to adjust treatment. NSUAD patients were more likely to fail on monotherapy, with almost 50% returning to their GP to either change the drug or to add an additional therapy. Monotherapy with INS proved to be inadequate for about 25% of SAR sufferers, an unexpectedly high proportion given their primacy of effect according to guidelines.^1,29,30,33^ More than 15% of patients initially prescribed multiple therapies also necessitated additional GP consultations for rhinitis-related motives. This suggests that current rhinitis treatment, including monotherapy and multiple therapies, does not meet patient clinical needs.

### Interpretation of findings in relation to previously published work

Such failure rates could be because AR is becoming more difficult to manage, with many patients presenting to their doctor with moderate/severe and persistent disease,^11,18^ with mixed disease (i.e., an allergic and non-allergic component)^22^ and with sensitivity to multiple allergens (i.e., polysensitisation), some of which may be inter-related.^4,20,21^ For many, INS monotherapy does not provide the expected level of symptom relief,^34^ highlighting the need for a more effective AR treatment option. Another possibility is that patients were not shown how to use their nasal spray properly,^33^ nor were they told that it requires regular use over several days before effects are noticed. One in five patients who receive guideline-directed care remain symptomatic with significant impairment to their quality of life, a phenotype known as severe chronic upper airway disease.^23^

Many SAR and NSUAD sufferers (33% and 23%, respectively) received a multi-therapy regimen as their initial prescription. OAH+INS was the most common co-prescription for both groups, but eye drops were also frequently added to OAH, INS and OAH+INS regimens. The high co-prescription rate at the start of both seasons was a further unexpected finding, which implies that these sufferers most likely were prescribed multiple therapies in previous seasons and thus started subsequent seasons on the same regimen. However, the high rate of additional consultations for multiple therapy starters suggests that symptom control was insufficient for these patients as well. Furthermore, the prevalence of eye drop addition to monotherapy and dual therapies (particularly for SAR) emphasises the burden of ocular symptoms for AR patients and the inadequacy of OAH and INS to effectively control them, a finding supported by others.^10,11^

### Strengths and limitations of this study

This large database survey included GP prescription data from 354 UK practices and information on 25,069 AR patients in 2009 and 22,381 in 2010. As it used the prescription data only, rather than patient-reported outcomes, it provides a unique insight into the burden of AR from the payer perspective. Over-the-counter medication use was not captured in the OPCRD. As patients frequently self-medicate, the true prevalence of multi-therapy use is likely to be much higher than that reported here.^35,36^ Patients in the database were ‘real-life’ patients, with a diagnosis of rhinitis routinely recorded by their GP, and are representative of the AR population as a whole in the UK. Assessment of data over two complete and consecutive seasons, with similar findings recorded, confirms the robustness of the data. A period broader than the pollen season defined in the literature was chosen to ensure that the first prescription of the season was captured.^37^ Categorisation of patients according to AR phenotype provided an insight into how seasonal and nonseasonal patients are managed in a GP setting. Higher co-prescribing behaviour and monotherapy failure observed for nonseasonal patients demonstrates the sensitivity of this database survey to quantify the burden of disease by AR phenotype. Finally, one of the strengths of the study is its observational nature, which allowed an overview of the current state of practice in the UK. Although lacking in precision, this approach provides insights into prescribing behaviour that could hardly be obtained with other approaches, as even patient or doctor surveys can act as an intervention, distorting behaviour.

A potential limitation of the survey was that no information was recorded on the method of AR diagnosis, the type of contact between GPs and patients when treatment was escalated or changed, or on the rationale for prescribing choices, which would have permitted a more precise assessment of concordance with guideline-recommended diagnosis procedure.^1,28^ Patients were also not categorised according to disease severity (i.e., mild and moderate/severe), which may have yielded interesting sensitivity findings. As AR patients frequently purchase medication over the counter without seeking professional help, it is possible that our sample was skewed towards more severe AR patients who experience symptoms that are debilitating enough to visit their GP.^35,36^ To capture the full population of patients with rhinitis, we included all Read codes on rhinitis, hay fever and AR ever recorded (Supplementary Table S2), as it was possible that these codes could be present only once in a patient’s medical history. We then used AR therapy prescriptions as a proxy for AR management. However, there is a possibility that scripts for the drugs classified as AR therapy were prescribed for another indication. Many patients were diagnosed with co-morbid asthma or eczema, but while asthma treatment may include systemic steroids, only a small proportion of patients in the current study received such prescriptions (4–7%). Furthermore, although eczema treatment may include OAH, it is more commonly managed with topical therapies.^38^ Finally, urticaria (which often does not require treatment; http://www.nhs.uk/conditions/nettle-rash/Pages/Introduction.aspx) and nasal polyps may be treated with OAH and INS, respectively, but the number of patients with these co-morbidities was relatively low (9–12% and 1–4% for urticaria and nasal polyps, respectively). Very few patients in this study received immunotherapy. This treatment is typically prescribed by allergists/specialists in the UK and thus captured infrequently in the database, thereby precluding an assessment of its role in seasonal and nonseasonal therapy. Although the data are specific to the UK, they do offer important insight to other countries, and add to the overall evidence of a high incidence of multiple therapy prescriptions, a trend that has been observed in many European countries,^39–42^ as well as in the United States.^43^

### Implications for future research, policy and practice

A shift to increased multi-therapy prescription was noted for both SAR and NSUAD patients, as the season progressed. As the number of therapies prescribed increased, so did the number of additional GP consultations. By season end, about 45% of SAR and over half of NSUAD sufferers were prescribed multiple therapies, not including over-the-counter medication usage. Although it is possible that patients changed treatment because of drug intolerance, the majority of patients received add-on therapy, suggesting that intolerance was not a major issue. Furthermore, to our knowledge, intolerance is not a major problem for non-sedating antihistamines and intranasal steroids used to treat AR. The in-season shift to multi-therapy use was most apparent in NSUAD. Prescribing increasing numbers of therapies from different AR medication classes would appear to be the logical response to monotherapy failure, increasing pathologic coverage and leading to improved symptom control. In the present survey, the most common treatment regimens at the end of the season was OAH+INS. However, this is neither recommended by the ARIA guidelines because of insufficient evidence,^1^ nor supported by evidence from the literature.^31,32^ Furthermore, this study found no evidence of improved outcomes in patients prescribed multi-therapy classes, as indicated by the frequent need for additional GP consultations in that group.

Our findings are consistent with the fact that the majority of moderate/severe AR patients are on multiple therapies in real life but continue to experience debilitating AR symptoms. Surveys assessing both prescription and over-the-counter medication use have shown co-medication behaviour in 66.0–74.4% of sufferers.^34,37,40,44^ These patients are constantly looking for new medications in an effort to find something that ‘works’,^45^ a treatment that can provide better and faster relief from both nasal and ocular symptoms.^37^ Disappointingly, patients continue to experience significant nasal and ocular symptom breakthrough despite treatment, even with multiple therapies,^11,39,46^ suggesting that there is an unmet pharmacologic need in AR.

A subgroup analysis in patients with co-morbid asthma (Supplementary Table S3) showed that SAR patients with an asthma diagnosis were less likely to be prescribed multiple therapies, both at the start and at the end of the season. This may reflect benefits of asthma treatment on rhinitis symptoms that have been previously described,^47,48^ and deserves to be explored in more detail in future studies.

### Conclusions

This study is the largest body of evidence investigating clinician prescribing behaviour in AR in the UK, and should be informative for both payers and prescribers. The high rate of monotherapy failure, the shift to multi-therapy prescription and the need for additional consultations during the season are useful proxy measures to assess unmet need. The extent of therapy failure and the rise in co-prescriptions during both seasons demonstrates that current therapy provides insufficient symptom relief for many patients and will increase the cost of AR management, both directly (drug costs) and indirectly (additional GP visits, absenteeism and presenteeism). Taken together, these findings support the need for novel AR treatment options that provide faster and more complete symptom control than current first-line therapies. However, as the majority of patients remained on their first medication, it is also vital to identify those who require more than one treatment at therapy initiation.

## Materials and methods

### Study design, study period and data collection

This was a prospective study of a historical cohort conducted with data from the Optimum Patient Care Research Database (OPCRD).^49^ The OPCRD is a quality-controlled, longitudinal, primary respiratory care database containing anonymous data from 354 general practices across the UK. The database contains information on patient management in primary and secondary care and combines electronic patient records with linked patient-reported data, which are collected using disease-specific questionnaires. These routine clinical data are extracted from practice management systems and include, for example, demographic characteristics, co-morbidities and current therapy.

Routine consultation data from electronic patient records, recorded by GPs at each patient visit, were used in this study. This included face-to-face consultations, telephone consultations and home visits. Diagnoses and prescription data were collected for both the 2009 and 2010 UK pollen seasons in order to assess consistency of findings. The season was defined as 1st March to 31st August, coinciding with the months in both years when most patients received an AR diagnosis or prescription.

The OPCRD has received approval for use in clinical research by the Trent Multi-Centre Research Ethics Committee (approval reference 10/H0405/3). Formal ethics and research management approval for this study were gained from the Anonymised Data Ethics Protocols and Transparency committee, which is the independent scientific advisory committee for the OPCRD.

### Patients

Included patients had a Read code-recorded diagnosis relating to hay fever or rhinitis at any time in the OPCRD (Supplementary Table S2), and had received at least one AR therapy prescription during the period from 1 March to 31 August 2009 and/or 2010. Recorded AR therapies included OAH, INS, non-steroidal nasal sprays, leukotriene receptor antagonists, topical ocular therapy for allergic conjunctivitis (eye drops) and systemic treatments, including systemic corticosteroids and immunotherapy. Non-steroidal nasal sprays included nasal antihistamines (24% of all prescriptions in this category) decongestants (oxymetazoline, xylometazoline and ephedrine), anticholinergics (ipratropium) and anti-inflammatory preparations (cromolyn and sodium cromoglicate). Furthermore, patients were categorised as having either SAR or receiving prescriptions for NSUAD. The latter proxy was designed to capture patients with perennial AR who probably account for the majority of the NSUAD group. Patients defined as SAR patients had no recorded AR treatment in the six months preceding the first prescription of the study period (i.e., symptoms were purely seasonal). Patients defined as NSUAD patients suffered from symptoms during the pollen season and outside it, as indicated by at least one AR therapy prescription in the six months preceding the first prescription of the study period. Patients with NSUAD who did not also experience a seasonal flare, as indicated by no prescription during the season, were thus excluded.

**Figure 3:**
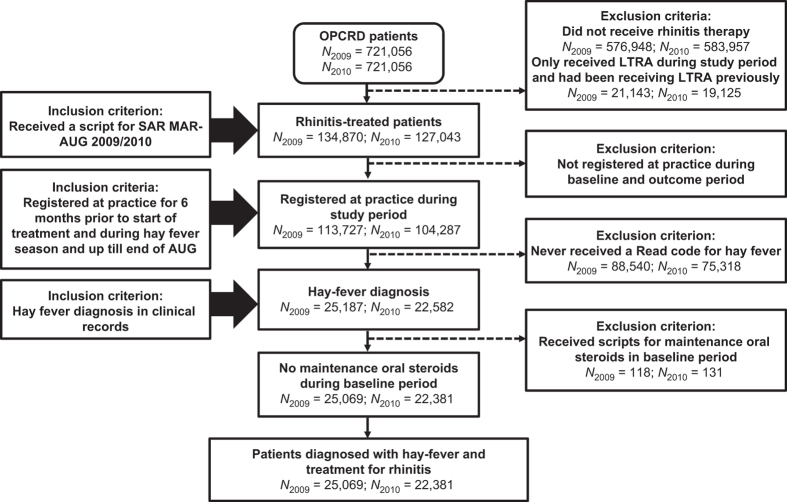
Consort Diagram. OPCRD, Optimum Patient Care Research Database. Patient data were taken from the OPCRD. Inclusion and exclusion criteria were applied as described in the text.

Patients receiving maintenance oral steroids during the six months before the study period were excluded, as long-term oral steroids may blunt SAR symptoms. Patients who received leukotriene receptor antagonists alone during the preceding 6 months were also excluded, as the leukotriene receptor antagonists may have been prescribed for the treatment of asthma rather than AR (Figure 3).

### Study end points

Socio-demographic information was collected, including patients’ age, gender and smoking history. Co-morbid diagnoses of asthma, urticaria, eczema and/or nasal polyps were also noted. Medication-related end points for the period from 1st March to 31st August for both 2009 and 2010 included the following:

- First recorded prescription of the season (i.e., monotherapy or multiple therapy).
- Treatment outcomes for patients who started the season on monotherapy. Outcomes were categorised into 4 groups: (i) stay on the same therapy; (ii) change drug; (iii) add on a new therapy; and (iv) change drug and add on a new therapy.
- Dynamics of prescription change during the season (i.e., proportion of patients on monotherapy and multiple therapy at season start and season end) and the proportion of each multiple therapy regimen by season end.
- Proportion of patients requiring additional consultations for rhinitis (i.e., where a rhinitis-related Read code was recorded) after the first consultation of the hay fever season, according to number of therapies prescribed at the beginning and during the season.

### Statistics

Data were analysed using IBM SPSS Statistics for Windows, Version 21.0 (Released 2012; IBM Corp., Armonk, New York, NY, USA). Descriptive statistics (mean, s.d.) were computed to summarise patient demographic and baseline characteristics. All other data are presented as number of patients and percentage of the population. Data are presented by AR phenotype (i.e., SAR or NSUAD) and by year (i.e., 2009 and 2010).

## Figures and Tables

**Table 1:** Patient demographic and baseline characteristics

	*2009 (*n*=25,069)*		*2010 (*n*=22,381)*	
	*SAR (*n*=18,341)*	*NSUAD (*n*=6,728)*	*SAR (*n*=16,187)*	*NSUAD (*n*=6,194)*
Age, years mean (s.d.)	31.3 (20.1)	46.5 (22.1)	32.1 (20.3)	47.7 (21.9)
Male, *n* (%)	8,811 (48.0)	2,636 (39.2)	7,679 (47.4)	2,426 (39.2)
				
*Recorded smoking status, *n* (%)*	15,406 (84.0)	6,397 (95.1)	13,266 (82.0)	5,873 (94.8)
Non-smokers	11,050 (71.7)	4,101 (64.1)	9,444 (71.2)	3,728 (63.5)
Current smokers	1,889 (12.3)	707 (11.1)	1,637 (12.3)	644 (11.0)
Ex-smokers	2,467 (16.0)	1,589 (24.8)	2,185 (16.5)	1,501 (25.5)
				
Asthma diagnosis, *n *(%)	7,145 (39.0)	3,745 (55.7)	5,517 (34.1)	3,270 (52.8)
Eczema diagnosis, *n* (%)	6,481 (35.3)	2,645 (39.3)	5,603 (34.6)	2,364 (38.2)
Urticaria, *n* (%)	1,621 (8.8)	809 (12.0)	1,553 (9.6)	596 (9.6)
Nasal polyps, *n* (%)	167 (0.9)	283 (4.2)	150 (0.9)	263 (4.2)

Abbreviations: NSUAD, nonseasonal upper airways disease; SAR, seasonal allergic rhinitis.

**Table 2:** Initial recorded prescription of the period 1st March-31st August

N *(%)*	*2009 (*n*=25,069)*		*2010 (*n*=22,381)*	
	*SAR (*n*=18,341)*	*NSUAD (*n*=6,728)*	*SAR (*n*=16,187)*	*NSUAD (*n*=6,194)*
*Monotherapy*				
OAH	9,505 (51.8)	3,378 (50.2)	8,495 (52.5)	3,108 (50.2)
INS	1,462 (8.0)	1,027 (15.3)	1,172 (7.2)	1,025 (16.6)
Systemic steroid	656 (3.6)	472 (7.0)	560 (3.5)	365 (5.9)
ED	559 (3.1)	251 (3.7)	472 (2.9)	219 (3.5)
Non-steroidal spray	68 (0.4)	35 (0.5)	40 (0.3)	35 (0.6)
LTRA	38 (0.2)	16 (0.2)	26 (0.2)	8 (0.1)
Immunotherapy	1 (0.01)	2 (0.03)	1 (0.01)	3 (0.1)
Total single therapy	12,289 (67.0)	5,181 (77.0)	10,766 (66.5)	4,763 (76.9)
				
*Multiple therapy*				
OAH+INS	2,153 (11.7)	676 (10.1)	1,882 (11.6)	622 (10.0)
OAH+ED	1,636 (8.9)	240 (3.6)	1,472 (9.1)	229 (3.7)
OAH+INS+ED	1,635 (8.9)	204 (3.0)	1,543 (9.5)	180 (2.9)
INS+ED	330 (1.8)	97 (1.4)	287 (1.8)	84 (1.4)
OAH+LTRA	27 (0.2)	128 (1.9)	14 (0.1)	116 (1.9)
Other multiple therapy	271 (1.5)	202 (3.0)	223 (1.4)	200 (3.2)
Total multiple therapy	6,052 (33.0)	1,547 (23.0)	5,421 (33.5)	1,431 (23.1)

Abbreviations: ED, eye drops; INS, intranasal corticosteroid; LTRA, leukotriene receptor antagonists; OAH, oral antihistamine; NSUAD, nonseasonal upper airways disease; SAR, seasonal allergic rhinitis.

**Table 3:** Dynamics of prescription changes during the hay fever season

N *(%)*	*2009 (*n*=25,069)*				*2010 (*n*=22,381)*			
	*SAR (*n*=18,341)*		*NSUAD (*n*=6,728)*		*SAR (*n*=16,187)*		*NSUAD (*n*=6,194)*	
	*Season start*	*Season end*	*Season start*	*Season end*	*Season start*	*Season end*	*Season start*	*Season end*
Monotherapy	12,289 (67.0)	10,136 (55.3)	5,181 (77.0)	3,130 (46.5)	10,776 (66.6)	8,850 (54.7)	4,764 (76.9)	2,974 (48.0)
Dual therapy	4,314 (23.5)	5,892 (32.1)	1,265 (18.8)	2,741 (40.7)	3,782 (23.4)	5,213 (32.2)	1,172 (18.9)	2,445 (39.5)
Triple therapy	1,717 (9.4)	2,243 (12.2)	272 (4.0)	755 (11.2)	1,615 (10.0)	2,062 (12.7)	244 (3.9)	694 (11.2)
4 therapies	20 (0.1)	68 (0.4)	10 (0.2)	92 (1.4)	24 (0.2)	62 (0.4)	14 (0.2)	77 (1.2)
5 therapies	1 (0.0)	2 (0.0)	0 (0.0)	9 (0.1)	0 (0.0)	0 (0.0)	0 (0.0)	4 (0.1)
Total multiple therapies	6,052 (33.0)	8,205 (44.7)	1,547 (23.0)	3,598 (53.5)	5,421 (33.5)	7,337 (45.3)	1,430 (23.1)	3,220 (52.0)
								
*Multiple therapies prescribed by season end*								
OAH+INS	2,938 (16.0)		1,448 (21.5)		2,590 (16.0)		1,278 (20.6)	
OAH+INS+ED	2,026 (11.0)		466 (6.9)		1,894 (11.7)		453 (7.3)	
OAH+ED	2,086 (11.4)		479 (7.1)		1,882 (11.6)		453 (7.3)	
INS+ED	385 (2.1)		143 (2.1)		335 (2.1)		127 (2.1)	
Other	770 (4.2)		1,062 (15.8)		636 (3.9)		909 (14.7)	

Abbreviations: ED, eye drop; INS, intranasal corticosteroid; NSUAD, nonseasonal upper airways disease; OAH, oral antihistamine; SAR, seasonal allergic rhinitis.
