# Supplementary material for: UK prescribing practices as proxy markers of unmet need in allergic rhinitis: a retrospective observational study
Source: NPJ Prim Care Respir Med. 2016 Jun 23;26:16033–. doi: 10.1038/npjpcrm.2016.33 (PMC4918055; doi:10.1038/npjpcrm.2016.33)
Supplement: Supplementary Information [file npjpcrm201633-s1.doc]

**Supplementary Information**

**Supplementary methods**

*Read codes*

Read code lists were generated by selecting a series of search terms based on clinical advice and literature research, which were used to compile the lists by searching version 2 of the NHS Read code browser. Where available, the lists were cross-checked with Quality and Outcomes Framework (QOF) Read code lists. The compiled Read code lists were reviewed by clinical advisers and sent for approval by the Anonymous Data Ethics Protocol and Transparency (ADEPT) committee, which is the independent scientific advisory committee for the Optimum Patient Care Research Database (OPCRD).

**Table S1** Therapy outcome for those allergic rhinitis patients starting the season on monotherapies

| First drug of the season | Stay on same therapy  N (%) | | Change drug  N (%) | | Add-on new therapy  N (%) | | Change drug & add-on new therapy N (%) | |
| --- | --- | --- | --- | --- | --- | --- | --- | --- |
|  | **Seasonal allergic rhinitis (SAR) patients** | | | | | | | |
|  | 2009 | 2010 | 2009 | 2010 | 2009 | 2010 | 2009 | 2010 |
| OAH | 7568 (79.6) | 6814 (80.2) | 835 (8.8) | 687 (8.1) | 1490 (15.7) | 1311 (15.4) | 388 (4.1) | 317 (3.7) |
| INS | 1133 (77.5) | 869 (74.1) | 58 (4.0) | 48 (4.1) | 312 (21.3) | 288 (24.6) | 41 (2.8) | 33 (2.8) |
| NS | 44 (64.7) | 30 (75.0) | 0 (0.0) | 0 (0.0) | 24 (35.3) | 10 (25.0) | 0 (0.0) | 0 (0.0) |
| ED | 374 (66.9) | 293 (62.1) | 15 (2.7) | 13 (2.8) | 184 (32.9) | 176 (37.3) | 14 (2.5) | 10 (2.1) |
| LTRA | 23 (60.5) | 23 (88.5) | 0 (0.0) | 0 (0.0) | 15 (39.5) | 3 (11.5) | 0 (0.0) | 0 (0.0) |
|  | **Non-seasonal upper airways disease (NSUAD) patients** | | | | | | | |
| OAH | 2025 (59.9) | 1945 (62.6) | 425 (12.6) | 331 (10.6) | 1165 (34.5) | 1012 (32.6) | 237 (7.0) | 180 (5.8) |
| INS | 554 (53.9) | 578 (56.4) | 84 (8.2) | 72 (7.0) | 448 (43.6) | 424 (41.4) | 59 (5.7) | 49 (4.8) |
| NS | 15 (42.9) | 14 (38.9) | 0 (0.0) | 0 (0.0) | 20 (57.1) | 22 (61.1) | 0 (0.0) | 0 (0.0) |
| ED | 95 (37.8) | 75 (34.2) | 17 (6.8) | 19 (8.7) | 155 (61.8) | 140 (63.9) | 16 (6.4) | 15 (6.8) |
| LTRA | 5 (31.3) | 3 (37.5) | 0 (0.0) | 0 (0.0) | 11 (68.8) | 5 (62.5) | 0 (0.0) | 0 (0.0) |

OAH: oral antihistamine; INS: intranasal corticosteroid; NS: non-steroidal nasal spray; ED: eye drop; LTRA: leukotriene receptor antagonist

*Some patients both changed drug and added a therapy therefore percentages do not add up to 100.*

2009: SAR n=12289; NSUAD n=5181; 2010: SAR n= 10766; NSUAD n=4763.

**Table S2** List of rhinitis and hay fever Read codes

| **RHINITIS CODES** | |
| --- | --- |
| **READ CODE** | **RED TERM** |
| H00..16 | Rhinitis – acute |
| H120.00 | Chronic rhinitis |
| H120000 | Chronic simple rhinitis |
| H120100 | Chronic catarrhal rhinitis |
| H120200 | Chronic hypertrophic rhinitis |
| H120300 | Chronic atrophic rhinitis |
| H120400 | Chronic infective rhinitis |
| H120500 | Chronic ulcerative rhinitis |
| H120600 | Chronic membranous rhinitis |
| H120700 | Chronic fibrinous rhinitis |
| H120z00 | Chronic rhinitis NOS |
| H17..11 | Perennial rhinitis |
| H18..00 | Vasomotor rhinitis |
| **ALLEGIC RHINITIS CODES** | |
| H17..00 | Allergic rhinitis |
| H171.00 | Allergic rhinitis – other allergens |
| H171.00 | Allergic rhinitis – unspecific allergen |
| H17z.00 | Allergic rhinitis NOS |
| Hyu2100 | [X]Other allergic rhinitis |
| **HAY FEVER CODES** | |
| H170.11 | Hay fever – pollens |
| H171.14 | Hay fever – other allergens |
| H172.11 | Hay fever – unspecific allergen |
| H170.00 | Allergic rhinitis pollens |
| Hyu2000 | [X]Other seasonal allergic rhinitis |

NOS = Not otherwise specified

**Table S3.** Dynamics of prescription changes during the hay fever season for patients with and without comorbid asthma

|  | | 2009 | | | | 2010 | | | |
| --- | --- | --- | --- | --- | --- | --- | --- | --- | --- |
|  |  | ASTHMA | | NON ASTHMA | | ASTHMA | | NON ASTHMA | |
|  |  | SAR  (n=7145) | NSUAD  (n=3745) | SAR  (n=11196) | NSUAD  (n=2983) | SAR  (n=5517) | NSUAD  (n=3270) | SAR  (n=10670) | NSUAD  (n=2924) |
| **Season Start** | Single therapy n  (%) | 5152  (72.1%) | 2859  (76.3%) | 7137  (63.7 %) | 2322  (77.8%) | 3,981  (70.5) | 2,490  (76.1) | 6,785  (63.6) | 2,274  (77.8) |
|  | Multiple therapies n  (%) | 1993  (27.9 %) | 886  (23.7%) | 4059  (36.3%) | 661  (22.2%) | 1,536  (24.6) | 780  (23.9) | 3,885  (36.4) | 650  (22.2) |
| **Season End** | Single therapy n  (%) | 4191  (58.7%) | 1665  (44.5%) | 5945  (53.1%) | 1465  (49.1 %) | 3 221  (58.4) | 1 465  (44.8) | 5 629  (52.8) | 1 509  (51.6) |
|  | Multiple therapies n  (%) | 2954  (41.3%) | 2080  (55.5%) | 5252  (46.9%) | 1518  (50.1%) | 2 296  (41.6) | 1 805  (55.2) | 5 041  (47.2) | 1 415  (48.4) |

SAR: seasonal allergic rhinitis; NSUAD: non-seasonal upper airways disease.
